# Preconception Health of Indigenous Peoples in Australia, Canada, New Zealand, and the United States: A Scoping Review

**DOI:** 10.3390/ijerph21030345

**Published:** 2024-03-14

**Authors:** Clara Walker, Tahmina Begum, Jacqueline A Boyle, James Ward, Federica Barzi

**Affiliations:** 1 UQ Poche Centre for Indigenous Health, The University of Queensland, Toowong, QLD 4066, Australia; 2 Centre for Health Research, University of Southern Queensland, Toowoomba, QLD 4350, Australia; 3 Eastern Health Clinical School, Monash University, Box Hill, VIC 3128, Australia

**Keywords:** preconception health, pre-pregnancy, Indigenous health

## Abstract

Background: There is increasing recognition of the importance of the preconception period for addressing reproductive and intergenerational health inequities and supporting improved maternal and child health outcomes. This study aimed to understand the extent and type of evidence that exists in relation to preconception health for Indigenous peoples living in high-income countries with similar experiences of colonisation, namely, Australia, New Zealand, Canada, and the United States. Methods: This review was conducted as per the JBI methodology and PRISMA Extension for Scoping Reviews. A comprehensive search of PubMed, CINAHL [EBSCO], Ovid Embase, Scopus, and the Wiley Cochrane Library was conducted using keywords and index terms. We included research in English published between January 2010 and June 2023 on quantitative and qualitative primary studies. Data were extracted using a standardised tool, and the analysis included quantitative descriptions and qualitative content analysis. Results: We identified 360 potential studies and included 57 articles in the review. Most studies were from the United States (n = 36, 63.2%) and Australia (n = 13, 22.8%), and they commonly reported associations between preconception health risk factors and maternal or child health outcomes (n = 27, 48.2%) or described the development, implementation, or evaluation of preconception health interventions (n = 26, 46.4%). Common preconception health areas were pre-pregnancy body mass index or weight (n = 34), alcohol (n = 16), diet (n = 14), physical activity (n = 12), and diabetes (n = 11). Most studies focused exclusively on women (n = 46, 80.7%), and very few included men (n = 3, 5.3%). The study populations were mostly urban and rural (n = 25, 43.9%) or rural only (n = 14, 24.6%); however, the geographical remoteness was often unclear (n = 14, 24.6%). Conclusions: While there was some research relating to the preconception health of Indigenous peoples, this review identified considerable research gaps. There is a need for dedicated research into preconception health risk factors and reproductive health outcomes, attitudes and awareness of preconception health, and preconception health interventions for Indigenous peoples.

## 1. Introduction

There is increasing recognition globally of the importance of preconception health in influencing reproductive health outcomes by agencies such as the World Health Organisation [1]. The health and behaviours of women and men prior to conception can influence fertility as well as maternal, infant, and child health outcomes. Additionally, early pregnancy is a critical period for foetal development; however, many women will be unaware that they are pregnant in the first few weeks of gestation and continue behaviours that are not recommended in pregnancy [2,3]. Several definitions of the preconception period exist, including the biological perspective (i.e., the weeks before embryo development), the individual perspective (i.e., when a conscious decision to conceive is made), and the public health perspective (i.e., longer periods of months or years to address health risk factors) [4]. Hill et al. extended this definition to include a life course perspective, which acknowledges that preconception health can be addressed throughout the life course and therefore includes groups such as adolescents and newly sexually active individuals [5].

A range of preconception risk factors have been identified for men and women. Women and men living with obesity have an increased risk of infertility [4], and women living with obesity further have an increased risk of gestational diabetes, pre-eclampsia, obstetric complications, congenital abnormalities, and their offspring living with obesity in childhood [6,7,8]. Tobacco, alcohol, and recreational drug use can also reduce fertility in men and women [2,4], and use by women during pregnancy contributes to the risk of obstetric complications, preterm birth, and a low birthweight [6]. Maternal alcohol consumption in the weeks prior to and during pregnancy is associated with Fetal Alcohol Spectrum Disorder and birth defects [6]. Sexually transmissible infections in women prior to and during pregnancy are also associated with adverse reproductive outcomes [9,10,11]. Other preconception factors that can influence reproductive health outcomes include maternal and paternal age, chronic diseases such as type 2 diabetes and hypertension, and environmental exposures [2,6,12]. In addition to physical factors, an individual’s preconception mental health status can influence pregnancy and birth outcomes [13].

Preconception care is defined as “counselling and the provision of biomedical, behavioural and social health interventions to optimise the health of women and their partners prior to pregnancy and improve health related outcomes for themselves and their children” [3]. A recent systematic review found a lack of high-quality clinical guidelines on preconception care and that content on recommended care varied and few recommendations were supported by high-quality evidence [14]. Common content areas identified in the review of preconception guidelines were family and reproductive life planning, physical activity, weight and nutrition, chronic and infectious diseases, smoking, alcohol and environmental exposures, and mental health.

Indigenous peoples living in Australia, Canada, New Zealand, and the United States of America share similar colonial histories, as well as contemporary experiences of intergenerational trauma, socioeconomic disadvantage, and racism and inadequate health services [15,16]. These experiences have led to similar inequities in relation to a higher prevalence of preconception health risk factors and adverse reproductive health outcomes [17,18,19,20,21,22,23]. These adverse outcomes are often exacerbated in rural Indigenous populations who can experience additional barriers to accessing quality, culturally appropriate health care [24]. Despite sharing similar experiences, there is considerable heterogeneity of Indigenous populations living within these four countries, with unique histories, languages, and cultural practices. Additionally, each country has a unique health system and approach to providing health services for Indigenous peoples [25].

Given the increasing acknowledgement of the importance of the preconception period and health for addressing reproductive and intergenerational health inequities, there is value in synthesising the available evidence relating to the preconception health of Indigenous peoples, including how Indigenous Australians understand preconception health, interventions to support Indigenous Australians’ preconception health, as well as the access to and uptake of preconception care. As such, the aim of this scoping review is to assess the extent of the literature relating to the preconception health of and preconception health interventions for Indigenous peoples living in Australia, New Zealand, Canada, and the United States.

## 2. Methods

### 2.1. Search Strategy and Selection Criteria

A scoping review was conducted as per the JBI methodology [26] and PRISMA Extension for Scoping Reviews (PRISMA-ScR) [27]. A scoping review is a transparent, rigorous, and structured method to synthesise and analyse published research and to identify evidence gaps [28].

A comprehensive search strategy was developed in PubMed and CINAHL [EBSCO] using a combination of keywords and index terms used in relevant previous reviews [29,30,31,32] (File S1). The search strategy was adapted for additional databases: Ovid Embase, Scopus, and the Wiley Cochrane Library.

This review included studies relating to the preconception health of Indigenous peoples living in Australia, New Zealand, the United States, and Canada, with a focus on the following:

- The association of preconception health risk factors with fertility, maternal health outcomes (i.e., during pregnancy, birth, and the postnatal period), and child health outcomes;
- Indigenous peoples’ understandings and awareness of preconception health;
- Interventions to support preconception health (including evidence on intervention development, implementation, and evaluation);
- Uptake of preconception health care and interventions, including factors that affect uptake of and access to preconception health care and interventions;
- Health professionals’ knowledge, awareness, attitudes, and behaviours relating to preconception health.

Studies were included if they focused on Indigenous peoples or reported results separately for Indigenous peoples. Indigenous peoples were included as recognised by each country and included Aboriginal and Torres Strait Islander peoples in Australia, First Nations, Inuit, and Metis peoples (also known as Aboriginal peoples) in Canada, Māori people in New Zealand, and American Indians and Alaska Natives in the United States. While the population of interest was Indigenous women and men, studies with other participant groups were included if the focus was on Indigenous preconception health (e.g., health professionals, elders, and community members). Given variations in definition of the preconception period and the exploratory aim of this review [4,5], the preconception period was defined by the study under review. Studies were excluded if data relating to the preconception period could not be disaggregated from data during or after pregnancy.

Dorney et al.’s [14] review of preconception clinical guidelines was used as a framework to identify preconception health risk factors that were eligible for inclusion. Research which focused on health risk factors more generally without specifying their relevance to the preconception period was not included. Research which reported the prevalence of a preconception health risk factor (and predictors of prevalence) without exploring the relationship with pregnancy, birth, or child health outcomes was excluded. Finally, while planned pregnancy is an important element of preconception health care [33,34], studies which focused exclusively on contraception were excluded, as this was not a focus of the review.

Quantitative (experimental, quasi-experimental, and observational) and qualitative primary research studies published in English from 2010 to 25 June 2023 were included. This timeframe was selected to ensure recency of findings and also aligns with an emerging global focus on preconception health [1]. Systematic and narrative reviews, commentaries, and clinical guidelines were excluded; however, the reference lists of relevant reviews were searched. Conference abstracts, study protocols, and dissertations were excluded.

Two reviewers (CW and TB) independently screened all citations, abstracts, and full-text data, with any disagreements about study selection resolved via discussion or in consultation with a third reviewer (FB). The reference list of all included sources of evidence was also screened for additional studies. 

### 2.2. Data Extraction and Analysis

Data were extracted using a standardised tool by one reviewer (CW) and verified by a second reviewer (FB). Information was extracted about study design, setting and population, the areas of preconception health focused on, and key results. Preconception health areas were reported if they were identified as a focus of the study or intervention for Indigenous peoples, regardless of whether data could be extracted for the purpose of this review. Where possible, the geographical remoteness of study participants was identified and classified into ‘urban’, ‘rural’, ‘urban and rural’, or ‘not stated/unclear’ using each country’s standard geographical remoteness classification system [35,36,37,38], or the authors’ description of remoteness was used if a study location was not named. For studies reporting associations between preconception health risk factors and reproductive health outcomes, the association that adjusted for the greatest number of confounders was reported; however, studies were not excluded if only unadjusted analysis for Indigenous peoples was presented.

Given the aim of the study and the broad scope of methodologies accepted, no quality assessment was performed on eligible studies.

The characteristics of the 57 included studies were summarised with frequencies and proportions using Microsoft Excel. Inductive content analysis was conducted on qualitative data to identify common themes across studies [39].

## 3. Results

### 3.1. Search Results

**Figure 1:**
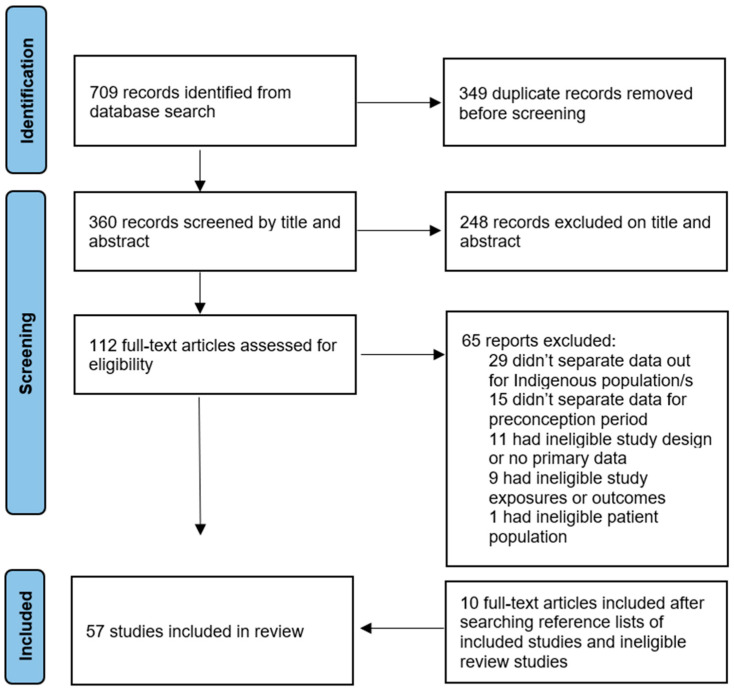
PRISMA diagram.

Electronic database searches identified 709 titles and abstracts. After removal of duplicates, 360 articles were screened by title and abstract, resulting in 112 potential articles for inclusion. After reviewing the articles’ full texts, 47 studies were included. An additional 10 studies were identified from reviewing the reference lists of the includes studies, leading to a total of 57 studies being included in this review (Figure 1).

### 3.2. Study Characteristics

Of the 57 studies identified, most were from the United States (36, 63.2%), followed by 13 from Australia (22.8%), 5 from Canada (8.8%), and 3 from New Zealand (5.3%) (Table 1).

Most studies were quantitative (n = 40, 70.2%), with common methodologies including retrospective cohort studies (n = 17, 29.8%), prospective cohort studies (n = 6, 10.5%), and cross-sectional studies (n = 5, 8.8%). Ten studies were qualitative (17.5%), commonly using interviews and/or focus groups, and seven were mixed methods (12.3%).

Studies mostly reported associations between preconception health risk factors and maternal or child health outcomes (n = 27, 48.2%) or described the development, implementation, or evaluation of preconception health interventions (n = 26, 46.4%) (Table 2 and Table 3). Fewer studies described Indigenous peoples’ uptake of preconception health care, including factors affecting the uptake of preconception health interventions and care, or Indigenous peoples’ or health professionals’ knowledge and attitudes towards preconception health (n = 4, 7.0%) (Table 4).

Most studies focused on one or two preconception health risk factors (n = 35, 61.4%) or three to four risk factors (n = 13, 22.8%), while several focused on five or more risk factors (5, 8.8%) or preconception health more generally (n = 4, 7.0%). Common preconception health areas of focus were pre-pregnancy BMI or weight (n = 34), alcohol (n = 16), diet (n = 14), physical activity (n = 12), pre-existing diabetes (n = 11), hypertension (n = 10), tobacco (n = 6), and folic acid (n = 6). Four studies included a focus on mental health.

Almost all study populations were exclusively women or young women (n = 46, 80.7%), while only two studies included men and women (3.5%). Eight studies (14.0%) included health professionals, elders or community members as participants, or a combination of these groups and women. One study included health professionals, men, and women (1.8%).

Study populations were mostly urban and rural (n = 25, 43.9%) or rural only (n = 14, 24.6%). Few studies were urban only (n = 4, 7.0%), and in a quarter of studies, the geographical remoteness of the population was not stated or unclear (n = 14, 24.6%).

### 3.3. Associations between Preconception Health Risk Factors and Maternal or Child Health Outcomes

Twenty-five studies focused on the association between pre-pregnancy BMI or weight (referred to hereafter as ‘pre-pregnancy BMI’) and a range of maternal and child health outcomes (Table 2) [10,41,42,46,47,49,50,51,53,55,56,60,62,64,66,67,72,73,75,85,86,91,93,94,95]. In general, these studies found associations between a higher pre-pregnancy BMI and adverse maternal and child health outcomes, including gestational diabetes [41,47,51,56,73,75], gestational hypertension [42,50,51], pre-eclampsia [93], caesarean section [51,93], high birthweight [51,55,67,72,85], and developing diabetes after pregnancy [53]. Reported relationships between pre-pregnancy BMI and gestational weight gain were not consistent across studies [49,55], and several studies found no association with a preterm birth [64,91] or an association with being underweight and preterm birth [51,60]. Similarly, some studies found no association between pre-pregnancy BMI and postpartum haemorrhage [46,66] or 3-year-old child weight [94].

Six studies also focused on associations between pre-pregnancy diabetes and maternal and child health outcomes [46,47,60,64,67,72]. Associations were reported between pre-pregnancy diabetes and preterm birth [47,60,64], postpartum haemorrhage [46], serious adverse events [47], caesarean section [47], and large-for-gestational-age/high-birthweight infants [47,72].

Two studies found associations with a history of depression pre-pregnancy [54] or a history of treatment or check-up for depression [82] and postpartum depression. One study found an association between chlamydia and/or gonorrhoea before pregnancy and miscarriage [10].

### 3.4. Studies Describing Development, Implementation, or Evaluation of Preconception Health Interventions

Eleven studies focused on the development, implementation, and/or evaluation of interventions to prevent alcohol-exposed pregnancy (Table 3) [45,63,65,68,69,70,71,74,77,78,79,87]. Quantitative evaluations of these interventions showed positive outcomes in reducing the risk of alcohol-exposed pregnancy in several studies [68,69,71] and no significant change between intervention and control groups in one study [78].

Eight studies focused on interventions to prevent gestational diabetes and therefore referred to BMI, healthy eating, and physical activity preconception health risk factors [80,81,83,88,89,92]. One quantitative study found that the mean knowledge and self-efficacy increased post-intervention [83], and another found an increase in certain healthy eating behaviours compared to the control group post-intervention [90]. Three studies focused on mandatory bread fortification with folic acid and/or iodine [40,57,58]. An Australian study found an increase in red cell folate and reduced Neural Tube Defects post-fortification [40], while a New Zealand study modelled that folic acid fortification would result in insufficient additional dietary folic acid [57]. Mandatory iodine fortification in New Zealand was found to reduce the proportion of women consuming insufficient iodine [58]. One intervention focused on improving individuals’ preconception diet and demonstrated a positive impact on Vitamin A and D consumption but no reduction in energy, sugar, or fat [52]. Another intervention focused on preconception health education more generally and found improvements in knowledge post-intervention [84].

A number of common themes emerged from qualitative data collection relating to the development, implementation, or evaluation of preconception health interventions. These included the role of family, community, and/or culture in promoting positive health behaviours [63,65,74,77,81,88,92]; the importance of culturally appropriate content [65,68,79,80,81,92]; the need for factual content on preconception health risks [65,80,88,89]; the need for effective communication with adolescents [74,81,88,92]; and the importance of a strength-based or empowerment approach [68,80].

### 3.5. Studies Describing Uptake of Preconception Care and Preconception Health Knowledge and Attitudes

Several studies reported on women’s access to preconception health information and/or care (Table 4) [44,76]. Other studies focused on specific topics or health conditions such as folic acid information [96] and women with heart disease receiving preconception counselling [59]. One study reported on young peoples’ knowledge and attitudes to preconception health and co-designed a resource that is available in print or electronically [43].

## 4. Discussion

This scoping review aimed to assess the extent of the literature relating to the preconception health of Indigenous peoples living in Australia, New Zealand, Canada, and the United States. The study identified a range of research which focused on the preconception health of Indigenous peoples, particularly from the United States and Australia. There was a range of evidence in some areas of preconception health, notably studies exploring associations between BMI and maternal health outcomes and, to a lesser extent, interventions focusing on a single preconception risk factor or outcome. There were notable differences in the areas of preconception health that was focused on by country, which may reflect variances in Indigenous populations as well as differing policy priorities. However, the review identified significant gaps in the literature relating to the preconception health of Indigenous peoples, including studies focusing on preconception health risk factors other than BMI and culturally appropriate preconception care interventions, as well as studies which focus on men as participants. Additionally, there was a lack of studies exploring Indigenous peoples’ knowledge and attitudes relating to preconception health, the uptake of preconception health interventions, and barriers and enablers to preconception care. Due to the variability in quantitative studies, including exposures, interventions, and outcomes, it was not possible to conduct any meta-analysis on the identified studies, and it is not recommended that a systematic review is conducted at this time.

Beyond the evidence relating to BMI and pre-pregnancy diabetes, the review found a lack of evidence on the associations between preconception health risk factors and maternal and child health outcomes. There are substantial risks associated with women who are either underweight or overweight prior to pregnancy, and as such, BMI is a focus of the broader preconception health epidemiology literature [4,97]. Indigenous women are overrepresented in both underweight and overweight categories, thereby warranting a strong focus on BMI as a preconception health risk factor [17,98]. Additionally, Indigenous women have a higher prevalence of pre-pregnancy diabetes, which is associated with adverse maternal and perinatal outcomes [23]. However, there are other preconception health risk factors which disproportionately affect Indigenous peoples and were missing from the epidemiological literature, such as tobacco use, physical inactivity, at-risk alcohol use, sexually transmissible infections, and psychological distress [19,20,22,99]. These are all factors that are described in various preconception guidelines [14] and have been linked to the ongoing impacts of colonisation and inequity in access to health care. Population-wide preconception health studies should be sufficiently powered where possible to facilitate stratified analysis of Indigenous participants. Studies should also disaggregate preconception and antenatal risk factors when collecting and analysing data, so that the relative impact of these two time periods can be better understood. Additionally, many studies were retrospective and thus often limited, by design, in terms of the availability of data on preconception care. Future studies should be prospective and specifically designed to gather direct and rigorous information on preconception care and preconception care services.

Given the cultural diversity of Indigenous peoples and identified barriers to accessing health care, there is a need for additional research into Indigenous peoples’ knowledge and attitudes relating to preconception health, uptake of preconception health interventions, and barriers and enablers to preconception care. These studies are important to inform the development of culturally appropriate interventions and policies to support Indigenous peoples’ preconception health. Similarly, there is a need for research to understand health professionals’ understandings and perspectives of delivering preconception care for Indigenous peoples to identify health system factors that support and hinder preconception care [100,101].

Despite the opportunity that the preconception period poses for improving maternal and child health outcomes, there remains a lack of interventions that can optimise preconception health factors for Indigenous peoples at an individual and population level across the life course. This is similar to the lack of preconception health interventions and high-quality evaluation of efficacy in the general population [102]. While this review identified a number of intervention studies, most focused on single preconception health factors, predominantly alcohol and folic acid/iodine fortification, as well as gestational diabetes prevention. There is benefit in designing and rigorously evaluating interventions for Indigenous peoples which address multiple preconception health risk factors in a holistic and culturally appropriate manner. The review identified a number of common themes relating to the development, implementation, or evaluation of preconception health interventions, including the role of family, community, and/or culture in promoting positive health behaviours [63,65,74,77,81,88,92]; the importance of culturally appropriate content [65,68,79,80,81,92]; and the importance of a strength-based or empowerment approach [68,80]. While these themes are supported by the broader literature on creating culturally appropriate health interventions for Indigenous peoples [103,104,105], preconception health interventions need to also ensure that Indigenous peoples are actively involved in every stage of program development and delivery. Additionally, interventions need to be holistic and based on a socio-ecological model that addresses the broader determinants of health at the individual, community, system, and policy levels. There is also value in prioritising the cultural determinants of health as part of an empowerment approach to optimising preconception health.

As with the literature on preconception health more generally [106], the identified studies almost all focused exclusively on women. Given the evidence that certain preconception health risk factors in men such as living with obesity and smoking can influence reproductive health outcomes [2,107], as well as the potential influence of men on their partners’ health [108], there is a need for a greater focus on men’s preconception health, including their knowledge on and attitudes to preconception health. Although this study did not focus primarily on fertility and infertility, there is a strong link between a range of preconception health risk factors and adverse fertility outcomes for both men and women [2,7]. Few studies reported fertility as a study outcome or content in preconception health education interventions. Future research on preconception health should consider including fertility as a reproductive health outcome, and qualitative research should also explore Indigenous peoples’ awareness of preconception health factors that influence fertility for men and women.

As with other social determinants of health, geographical remoteness is an important predictor of health outcomes in the countries studied [109]. While rural and remote populations are more likely to experience adverse health outcomes, health inequities persist in increasingly urbanised Indigenous populations [24]. However, many of the studies included in this review did not specify the geographical remoteness of participants, and rurality was infrequently included in analysis. A greater focus on the rurality of participants would assist in understanding the relative importance of geographical rurality in preconception health and health care access, and information on the geographical context of interventions may assist with the transferability of interventions.

As an exploratory scoping review, this study had several limitations. Firstly, the broad inclusion criteria led to some challenges in the study selection; however, any discrepancies between reviewers were resolved through discussion and, when necessary, a third reviewer. Nevertheless, it was necessary to place some limitations on the types of studies included, which may have skewed the study results. For example, only studies which separated out data on Indigenous peoples were included, which meant that New Zealand studies which combined Māori and Pasifika populations, and Hawaii-based studies which combined Native Hawaiian and other Pacific Islander populations, were excluded.

Similarly, studies which combined data on preconception and pregnancy risk factors were excluded, leading to the exclusion of studies focusing on key preconception risk factors such as tobacco, alcohol, and other drugs and mental health. Finally, studies which only reported the prevalence of a preconception risk factor without exploring the relationship with pregnancy, birth, or child health outcomes were excluded. These studies are a useful component of the evidence relating to preconception health, although data on the prevalence of many of these preconception health risk factors in Indigenous populations of reproductive age are available in government publications [19,20,21,110].

Additionally, the study did not review grey literature, which may have led to some preconception interventions being missed. Nevertheless, the study provides the first ever review of the preconception health of Indigenous peoples and identifies a range of priorities for further research on this important topic.

## 5. Conclusions

In conclusion, this study identified some evidence and considerable gaps in the literature relating to the preconception health of Indigenous peoples living in Australia, New Zealand, Canada, and the United States. Given the increasing acknowledgement of the importance of the preconception period for addressing reproductive and intergenerational health inequities, and for improving maternal and child health outcomes, there is a need for dedicated research into the preconception health risk factors and reproductive health outcomes, attitudes towards and awareness of preconception health, and preconception health interventions for Indigenous peoples.

## Figures and Tables

**Table 1:** Summary of included studies.

Author and Date	Indigenous Population	Geographical Remoteness	Study Type	Participants	Preconception Health Areas
Australia					
Bower et al. (2016) [40]	Aboriginal	Urban and rural	Repeated cross-sectional study	Men and women	Folic acid
Campbell et al. (2011) [10]	Aboriginal and Torres Strait Islander	Rural	Prospective cohort	Women	Tobacco, diet, BMI, waist circumference, physical activity, alcohol, diabetes, STIs, systolic and diastolic blood pressure (SBP and DBP), cholesterol, triglycerides, red cell folate, fasting glucose, albumin/creatinine ratio, gamma-glutamyl transferase, metabolic syndrome
Campbell et al. (2012) [41]	Aboriginal and Torres Strait Islander	Rural	Prospective cohort	Women	Tobacco, diet, BMI, waist circumference, physical activity, alcohol, diabetes, SBP and DBP, cholesterol, triglycerides, red cell folate, fasting glucose, albumin/creatinine ratio, gamma-glutamyl transferase, hyper-triglyceridemic waist, metabolic syndrome
Campbell et al. (2013) [42]	Aboriginal and Torres Strait Islander	Rural	Prospective cohort	Women	Tobacco, diet, BMI, waist circumference, physical activity, alcohol, SBP and DBP, cholesterol, triglycerides, red cell folate, fasting glucose, albumin/creatinine ratio, gamma-glutamyl transferase, metabolic syndrome
Gilbert et al. (2021) [43]	Aboriginal and Torres Strait Islander	Rural	Mixed methods	Men and women	General preconception health and fertility
Griffiths et al. (2020) [44]	Aboriginal	Rural	Retrospective audit	Women	Tobacco, diet, BMI, alcohol, mental health, STIs, proteinuria, folic acid, vaccination, cervical screening, chronic disease management, fertility management, family and domestic violence
Kennedy et al. (2021) [45]	Aboriginal and Torres Strait Islander	Urban and rural	Mixed methods	Women	Tobacco, diet, alcohol, mental health
Lucas et al. (2021) [46]	Indigenous Australians	Rural	Prospective cohort	Women	BMI, diabetes
Maple-Brown et al. (2019) [47]	Indigenous Australians	Rural	Prospective cohort	Women	BMI, diabetes
Munns et al. (2016) [48]	Aboriginal	Rural	Mixed methods	Health service staff	General preconception health
Schumacher et al. (2019) [49]	Indigenous Australians	Rural	Prospective cohort	Women	BMI, diabetes, hypertension
Sina et al. (2014) [50]	Aboriginal	Rural	Nested case–control	Women	BMI, SBP and DBP, pre-existing albuminuria, waist circumference, hip circumference, waist-to-hip ratio, waist-to-height ratio
Thrift and Callaway (2014) [51]	Indigenous Australians	Urban and rural	Retrospective cohort	Women	BMI, diabetes, hypertension
Canada					
Bains et al. (2014) [52]	Inuit and Inuvialuit	Rural	Controlled pre–post	Women	Diet, physical activity
Mackenzie et al. (2021) [53]	First Nations	Urban and rural	Retrospective cohort	Women	BMI ^#^, diabetes, hypertension
Nelson et al. (2018) [54]	First Nations, Inuit, and Métis	Urban and rural	Cross-sectional survey	Women	Mental health
Oliveira et al. (2013) [55]	First Nations	Urban	Retrospective cohort	Women	Tobacco, BMI, diabetes, hypertension
Oster et al. (2014) [56]	First Nations	Urban and rural	Retrospective cohort	Women	BMI ^#^, diabetes, hypertension
New Zealand					
Mallard et al. (2012) [57]	Māori	Not stated/unclear	Cross-sectional survey	Women	Folic acid
Mallard and Houghton (2014) [58]	Māori	Not stated/unclear	Cross-sectional survey	Women	Iodine
Tromop-van Dalen et al. (2021) [59]	Māori	Not stated/unclear	Mixed methods	Health professionals and women	Congenital and rheumatic heart disease
United States					
Anderson et al. (2016) [60]	American Indian and Alaska Native (AI/AN)	Urban and rural	Retrospective cohort	Women	BMI
Charron-Prochownik et al. (2023) [61]	AI/AN	Urban and rural	Randomised controlled trial (baseline only)	Women	BMI, physical activity, diet
Declercq et al. (2015) [62]	AI/AN	Urban and rural	Retrospective cohort	Women	BMI
Deutsch et al. (2021) [63]	AI/AN	Not stated/unclear	Qualitative written responses	Women	Alcohol
Dorfman et al. (2015) [64]	AI/AN	Urban and rural	Retrospective cohort	Women	BMI, diabetes, hypertension
Gorman et al. (2013) [65]	AI/AN	Not stated/unclear	Qualitative focus groups and interviews	Women and health service staff	Alcohol
Hadley et al. (2021) [66]	Alaska Native	Urban and rural	Case–control	Women	BMI
Halvorson et al. (2017) [67]	Native American	Urban and rural	Retrospective cohort	Women	BMI, diabetes
Hanson et al. (2011) [68]	American Indian	Not stated/unclear	Mixed methods	Elders, community, and women	Alcohol
Hanson et al. (2013) [69]	American Indian	Rural	Longitudinal survey	Women	Alcohol
Hanson et al. (2015) [70]	American Indian	Rural	Mixed methods	Women	Alcohol
Hanson et al. (2017) [71]	American Indian	Urban and rural	Longitudinal survey	Women	Alcohol
Hiratsuka et al. (2022) [72]	AI/AN	Urban and rural	Retrospective cohort	Women	BMI, diabetes
Hunsberger et al. (2010) [73]	AI/AN	Urban and rural	Retrospective cohort	Women	BMI
Jensen et al. (2016) [74]	American Indian	Urban and rural	Qualitative interviews	Health professionals, men, and women	Alcohol
Kim et al. (2013) [75]	American Indian	Urban and rural	Retrospective cohort	Women	BMI
Lammers et al. (2017) [76]	Native American	Rural	Cross-sectional survey	Women	General preconception health
Lowrey et al. (2019) [77]	American Indian	Urban and rural	Qualitative written responses	Women	Alcohol
Montag et al. (2015) [78]	AI/AN	Not stated/unclear	Randomised controlled trial	Women	Alcohol
Montag et al. (2017) [79]	AI/AN	Not stated/unclear	Qualitative focus groups and interviews	Health professionals, women, community members	Alcohol
Moore et al. (2019) [80]	AI/AN	Urban	Qualitative focus groups	Women	BMI, diet, physical activity
Moore et al. (2021) [81]	AI/AN	Not stated/unclear	Qualitative focus groups	Health professionals and elders	BMI, diet, physical activity
Mukherjee et al. (2018) [82]	AI/AN	Urban and rural	Retrospective cohort	Women	Mental health
Nadeau et al. (2020) [83]	AI/AN	Urban	Pre–post	Women	BMI, diet, physical activity
Richards and Mousseau (2012) [84]	American Indian	Not stated/unclear	Randomised controlled trial	Women	General preconception health
Rockhill et al. (2015) [85]	AI/AN	Urban and rural	Retrospective cohort	Women	BMI
Seal and Broome (2013) [86]	American Indian	Not stated/unclear	Cross-sectional survey	Women	BMI
Shreshtha et al. (2018) [87]	American Indian	Urban and rural	Mixed methods	Women	Alcohol
Stotz et al. (2019) [88]	AI/AN	Urban	Qualitative interviews and focus groups	Women	BMI, diet, physical activity
Stotz et al. (2021) [89]	AI/AN	Not stated/unclear	Qualitative interviews and focus groups	Health professionals, elders, and women	BMI, diet, physical activity
Stotz et al. (2023) [90]	AI/AN	Not stated/unclear	Randomised controlled trial	Women	BMI, diet, physical activity
Tabet et al. (2017) [91]	AI/AN	Urban and rural	Retrospective cohort	Women	BMI
Terry et al. (2020) [92]	AI/AN	Urban and rural	Qualitative interviews and focus groups	Health professionals	BMI, diet, physical activity
Tiwari et al. (2021) [93]	AI/AN	Not stated/unclear	Retrospective cohort	Women	BMI
Wojcicki et al. (2015) [94]	Alaska Native	Urban and rural	Retrospective cohort	Women	BMI
Zamora-Kapoor et al. (2016) [95]	AI/AN	Urban and rural	Retrospective cohort	Women	BMI

^#^ Studies used weight rather than BMI as a variable but included in the broader BMI category for analysis.

**Table 2:** Studies on associations between preconception health risk factors and maternal or child health outcomes.

Author and Date	Setting (Indigenous Population, Geographical Remoteness)	Study Description	Participants/Sample	Confounding Factors Adjusted For	Key Results
**Australia**					
Campbell et al. (2011) [10]	Aboriginal and Torres Strait Islander, Rural	Prospective cohort study of Indigenous women to understand predictors of miscarriage	Included 1009 Indigenous women of 15–44 years who participated in a 1999–2000 health screening program in far-north Queensland	Age and ethnicity Age, ethnicity, and risky drinking	APR of becoming pregnant: women who smoked vs. women who did not smoke: 1.38, 95% CI 1.13–1.69; women who at baseline had low red cell folate (<295 nmol/L) vs. not low red cell folate: 1.33, 95% CI 1.10–1.60. Other preconception health risk factors were not associated with becoming pregnant.APR of miscarriage: pre-pregnancy chlamydia vs. no chlamydia: 2.24, 95% CI 1.26–3.99; pre-pregnancy gonorrhoea vs. no gonorrhoea: 3.07, 95% CI 1.80–5.24; pre-pregnancy gonorrhoea and chlamydia vs. no STIs: 4.57, 95% CI 2.21–9.46. Other preconception health risk factors were not associated with miscarriage.
Campbell et al. (2012) [41]	Aboriginal and Torres Strait Islander, Rural	Prospective cohort study of Indigenous women to understand predictors of diabetes in pregnancy	Included 1009 Indigenous women of 15–44 years who participated in a 1998–2000 health screening program in north Queensland	Age and ethnicity	APR of diabetes in pregnancy: waist circumference, 1 cm: 1.04, 95% CI 1.01–1.06; BMI, 1 kg/m^2^: 1.06, 95% CI 1.02–1.11; SBP, 1 mm/Hg: 1.03, 95% CI 1.01–1.05; DBP, 1 mmHg: 1.03, 95% CI 1.00–1.07; fasting glucose, 1 mmol/L: 1.55, 95% CI 1.35–1.76; albumin/creatine ratio, 1 mg/mmol: 1.01, 95% CI 1.00–1.01; hyper-triglyceridemic waist vs. not HTW: 3.23, 95% CI 1.53–6.84; metabolic syndrome vs. not metabolic syndrome: 3.50, 95% CI 1.54–8.00. Other preconception health risk factors were not associated with diabetes in pregnancy.
Campbell et al. (2013) [42]	Aboriginal and Torres Strait Islander, Rural	Prospective cohort study of Indigenous women to understand predictors of hypertension in pregnancy	Included 1009 Indigenous women of 15–44 years who participated in a 1998–2000 health screening program in north Queensland	Age and ethnicity	APR of hypertension in pregnancy: waist circumference, 1 cm: 1.04, 95% CI 1.02–1.06; BMI, 1 kg/m^2^: 1.09, 95% CI 1.04–1.14; SBP, 1 mmHg: 1.06, 95% CI 1.03–1.09; DBP, 1 mmHg: 1.07, 95% CI 1.03–1.11; metabolic disease vs. no metabolic disease: 3.50, 95% CI 1.50–8.17; red cell folate, 1 nmol/L: 1.00, 95% CI 1.00–1.01; albumin/creatinine ratio, 1 mg/mmol: 1.01, 95% CI 1.00–1.02; gamma-glutamyl transferase, 1 U/L: 1.01, 95% CI 1.00–1.02. Other preconception health risk factors were not associated with hypertension in pregnancy.
Lucas et al. (2021) [46]	Indigenous Australians, Rural	Prospective cohort study of women to understand risk factors for postpartum haemorrhage	Included 1102 women, including 525 Indigenous women in the Northern Territory, who participated in the Pregnancy And Neonatal Diabetes Outcomes in Remote Australia (PANDORA) study 2011–2017	Age	AOR of postpartum haemorrhage: women with type 2 diabetes vs. women with normal glycaemic tolerance: 1.91, 95% CI 1.07–3.41. BMI was not associated with postpartum haemorrhage (AOR 1 kg/m^2^: 1.01, 95% CI 0.98–1.03).
Maple-Brown et al. (2019) [47]	Indigenous Australians, Rural	Prospective cohort study of women to understand pregnancy outcomes associated with pre-pregnancy and gestational diabetes.	Included 1135 women, including 542 Indigenous women in the Northern Territory, who participated in the Pregnancy And Neonatal Diabetes Outcomes in Remote Australia (PANDORA) study 2011–2017	Crude	Women with type 2 vs. women without type 2 diabetes: rate of preterm birth: 38.8% vs. 5.1%, *p* < 0.001; rate of serious adverse events: 49.0% vs. 11.1%, *p* < 0.001; rate of caesarean sections: 71.4 vs. 27.3%, *p* < 0.001; rate of large-for-gestational-age babies: 39.5% vs. 10.3%, *p* < 0.001); mean (SD) pre-pregnancy BMI: 31.3 kg/m^2^ (SD 7.3) vs. 24.8 kg/m^2^ (SD 6.0) (*p* value not available).
Sina et al. (2014) [50]	Aboriginal, Rural	Nested case–control study of women living in a remote Aboriginal community to understand predictors of gestational hypertensive disorders	Included 168 women living in a remote Aboriginal community who participated in a health screening program from 1992 to 1995 and were subsequently pregnant (28 women with gestational hypertensive disorders, matched at baseline with 140 women)	Age at pregnancy, alcohol consumption, and smoking	AOR of gestational hypertensive disorders: BMI, 1 SD: 1.68, 95% CI 1.10–2.58; waist circumference, 1 SD: 1.78, 95% CI 1.10–2.89. There was no association between hip circumference, waist-to-hip ratio, or waist-to-height ratio and gestational hypertensive disorders.
Schumacher et al. (2019) [49]	Indigenous Australians, Rural	Prospective cohort study of pregnant women who identified as Indigenous or carrying an Indigenous child to understand the relationship between pre-pregnancy BMI and gestational weight gain	Included 110 pregnant women who identified as Indigenous or carrying an Indigenous child who attended antenatal clinics in regional and remote NSW	Crude	Excessive weight gain during pregnancy by BMI category: obese: 31%; overweight: 65%; healthy weight: 39%. Inadequate weight gain by BMI category: obese: 44%; overweight: 13%; healthy weight: 33%.
Thrift and Callaway (2014) [51]	Indigenous Australians, Urban and rural	Retrospective cohort study of women, including Indigenous women, to understand associations between overweight/obesity, Indigenous status and perinatal outcomes	Included 265,848 women, including 13,582 Indigenous women, with singleton births from July 2007 to 2011	Maternal age, parity, geographical location, and smoking	APR of gestational diabetes: overweight vs. normal weight women: 1.94, 95% CI 1.58–2.38; obese (BMI 30–39.9) vs. normal weight women: 3.00, 95% CI 2.47–3.63; severely obese (BMI 40+) vs. normal weight women: 4.44, 95% CI 3.48–5.67. APR of hypertensive disorders of pregnancy: overweight vs. normal weight women: 1.44, 95% CI 1.18–1.75; obese vs. normal weight women: 2.00, 95% CI 1.64–2.43, APR; severely obese vs. normal weight women: 2.12, 95% CI 1.53–2.94. APR of caesarean section: overweight vs. normal weight women: 1.18, 95% CI 1.08–1.29; obese vs. normal weight women: 1.32, 95% CI 1.21–1.44; severely obese vs. normal weight women: 1.81, 95% CI 1.58–2.07. APR of high birthweight (4000+g): overweight vs. normal weight women: 1.70, 95% CI 1.46–1.99; obese vs. normal weight women: 2.36, 95% CI 2.03–2.75; severely obese vs. normal weight women: 3.26, 95% CI 2.64–4.03. APR of preterm birth (<37 weeks): underweight vs. normal weight women: 1.98, 95% CI 1.36–2.88. APR of births < 32 weeks: underweight vs. normal weight women: 1.98, 95% CI 1.36–2.88 APR of low birthweight (<2500 g); underweight vs. normal weight women: 1.56, 95% CI 1.31–1.86. APR of 1-min Apgar scores < 7: obese vs. normal weight women: 1.23, 95% CI 1.06–1.42; severely obese vs. normal weight women:1.62, 95% CI 1.28–2.05. APR of 5-min Apgar score < 7: underweight vs. normal weight women: 1.71, 95% CI 1.15–2.54; severely obese vs. normal weight women: 1.85, 95% CI 1.18–2.88.
**Canada**					
Mackenzie et al. (2021) [53]	First Nations, Urban and rural	Retrospective cohort study of linked data of First Nations and non-First Nations women to understand risk of developing diabetes after pregnancy	Included 370,923 women, including 21,215 First Nations women, who gave birth in Alberta (singleton pregnancy) from 1999 to 2014	Maternal age, smoking anytime during pregnancy, pre-existing and pregnancy-induced hypertension, caesarean section, and stillbirth	HR of developing diabetes after pregnancy: First Nation women without GDM and <91 kg pre-pregnancy vs. non-First Nations women without GDM and <91 kg: 2.9, 95% CI 2.6–3.3; First Nations women without GDM and >=91 kg pre-pregnancy vs. non-First Nations women without GDM and <91 kg: 9.9, 95% CI 8.4–11.6; First Nations women with GDM and <91 kg pre-pregnancy vs. non-First Nations women without GDM and <91 kg: 30.9, 95% CI 26.2–36.4; First Nations women with GDM and >=91 kg vs. non-First Nations women without GDM and <91 kg: 54.4, 95% CI 45.0–65.7.
Nelson et al. (2018) [54]	First Nations, Inuit, and Métis, Urban and rural	Cross-sectional survey of Canadian women, including Aboriginal women, to understand determinants of postpartum depression	Included 76,508 women (weighted), including 3130 Aboriginal women (Inuit, Metis, or First Nations living off-reserve) who had given birth to a singleton live baby from November 2005 to May 2006	Age, marital status, education, employment, income, parity, time to prenatal care, planned pregnancy status, alcohol use, drug use, and smoking status during pregnancy	ARR of postpartum depression: First Nations women living off-reserve with a history of depression vs. no history of depression: 4.4, 95% CI 1.4–8.6. Metis women with a history of depression vs. no history of depression: 0.4, 95% CI 0.2–0.7. This could not be calculated for Inuit women due to small sample size.
Oliveira et al. (2013) [55]	First Nations, Urban	Retrospective cohort study of First Nations women compared with non-First Nations women to understand maternal profiles and birth outcomes	Included 451 First Nations women from the Six Nations Reserve, Ontario, with singleton pregnancies 2005–2010 and 622 non-First Nations women from nearby Hamilton, Ontario, with singleton pregnancies 2005–2009	CrudeNewborn sex, gestational age, hypertension prior to or during pregnancy, gestational weight gain, and smoking during pregnancy	Mean gestational weight gain (kg): normal weight: 16.5 kg (SD 8.5); overweight: 15.4 kg (SD 6.8); obese: 12.1 kg (SD 8.8). (difference between groups *p* < 0.001).Newborn birthweight (g): for each pre-pregnancy additional 1 kg/m^2^ BMI (e.g., 27 kg/m^2^ vs. 26 kg/m^2^): +17.8 g, 95% CI 9.6–26.1, *p* < 0.001.
Oster et al. (2014) [56]	First Nations, Urban and rural	Retrospective cohort study of First Nations and non-First Nations women to understand diabetes in pregnancy (pre-pregnancy and gestational diabetes)	Included 427,058 women, including 28,306 First Nations women, who gave birth in Alberta 2000–2009	Age, previous stillbirth, previous caesarean section, previous LGA infant, proteinuria, and drug dependency	AOR of gestational diabetes women with pre-pregnancy weight 91 kg+ vs. women <91 kg: 2.93, 95% CI 2.56–3.33.
**United States**					
Anderson et al. (2016) [60]	AI/AN, Urban and rural	Retrospective cohort study of birth data from women, including AI/AN women, to understand relationship between pre-pregnancy BMI, diabetes (pre-pregnancy and gestational), and birth outcomes	Included 5,193,386 women, including 44,570 AI/AN women, with singleton first births from 2009 to 2013	Age, education, calendar year, prenatal care, marital status, and child sex	OR of preterm birth: underweight vs. normal weight women: 1.20, 95% CI 1.03–1.38; women with pre-pregnancy diabetes vs. without diabetes (pre-pregnancy or gestational): 1.65, 95% CI 1.32–2.06.
Declercq et al. (2015) [62]	AI/AN, Urban and rural	Retrospective cohort study of women, including AI/AN women, to understand the relationship between pre-pregnancy obesity and caesarean delivery	Included 2,233,144 women, including AI/AN women, who had a singleton, vertex birth (37–41 weeks) in 2012 and no prior caesarean	Crude	Caesarean rates by BMI category: underweight: 11.2%; normal weight: 11.6%; overweight: 15.2%; obese I: 18.1%; obese II: 21.8%; obese III: 29.8%. No statistical analysis was performed on these variables for AI/AN women.
Dorfman et al. (2015) [64]	AI/AN, Urban and rural	Retrospective cohort study of women, including AI/AN women, to understand association between diabetes (pre-pregnancy and gestational) and preterm birth	Included 12,420 AI/AN women with live singleton births who participated in the 2004–2011 Pregnancy Risk Assessment Monitoring System (PRAMS)	Crude	OR of preterm birth: women with pre-pregnancy diabetes vs. without diabetes: 2.10, 95% CI 1.31–3.37; women with pre-pregnancy hypertension vs. no pre-pregnancy hypertension: 1.89, 95% CI 1.49–2.40. There was no association between pre-pregnancy BMI and preterm birth.
Hadley et al. (2021) [66]	Alaska Native, Urban and rural	Case–control study of Alaska Native women to understand risk factors for postpartum haemorrhage	Included 384 Alaska Native women (128 cases, 256 controls) who gave birth in 2018–2019 at the Alaska Native Medical Center	Crude	OR of postpartum haemorrhage: women with BMI 40+ vs. BMI <40: 2.6, 95% CI 1.4–4.5. There was no association in the adjusted analysis.
Halvorson et al. (2017) [67]	Native American, Urban and rural	Retrospective cohort study of birth data from women, including Alaska Native women, to understand relationship between race, pre-pregnancy BMI, diabetes, and birth outcomes	Included 70,679 women, including 11,416 Native American women with singleton live births from 2006 to 2011	Crude	Mean birthweight by diabetes status: women with pre-pregnancy diabetes: 3524 g; women without diabetes: 3366 g; women with gestational diabetes: 3544 g. No statistical analysis was performed on these variables.Mean birthweight by BMI category: underweight: 3210 g; normal weight: 3311 g; overweight: 3416 g; obese: 3463 g. No statistical analysis was performed on these variables.
Hiratsuka et al. (2022) [72]	AI/AN, Urban and rural	Retrospective cohort study of AI/AN women to understand the association of macrosomia with pre-pregnancy health status and social determinants of health	Included 1136 AI/AN women with singleton births from 2011–2019 receiving Indian Health Service care	Age at pregnancy, health insurance type, chronic conditions, gestation, and country-level social determinants of health indicators	AOR of macrosomia: overweight vs. underweight/normal weight women: 2.81, 95% CI 1.05–7.51; obese vs. underweight/normal weight women: 3.43, 95% CI 1.33–8.87; women with pre-pregnancy diabetes vs. no diabetes: 10.31, 95% CI 4.19–25.37; women living in rural counties vs. urban counties: 4.01, 95% CI 1.02–15.70.
Hunsberger et al. (2010) [73]	AI/AN, Urban and rural	Retrospective cohort study of Oregon women, including AI/AN women, to understand risk factors for gestational diabetes	Included 3883 women, including 493 AI/AN women, with live births who participated in the 2004–2011 Pregnancy Risk Assessment Monitoring System (PRAMS)	Crude	Prevalence of gestational diabetes by BMI category: BMI > 26: 10.3%; BMI <= 26: 4.7%. No statistical analysis was performed on these variables.
Kim et al. (2013) [75]	American Indian, Urban and rural	Retrospective cohort study of women, including Native American women, to understand contribution of BMI to gestational diabetes risk across racial groups	Included 1,228,265 women, including 4134 American Indian women, with a live singleton birth in 2007–2009	Maternal age, parity, and nativity	ARR of gestational diabetes: overweight vs. normal weight women: 2.2, 95% CI 1.6–3.1; class I obese vs. normal weight women: 3.1, 95% CI 2.2–4.3; class II obese vs. normal weight women: 4.2, 95% CI 2.9–6.1; class III obese vs. normal weight women: 5.7, 95% CI 4.0–8.2.
Mukherjee et al. (2018) [82]	AI/AN, Urban and rural	Retrospective cohort study of women, including AI/AN women, to understand risk factors for postpartum depression	Included 87,565 women, including 2757 AI/AN women, who participated in the 2009–2011 Pregnancy Risk Assessment Monitoring System (PRAMS)	Maternal education, marital status, pregnancy intention, and prenatal stressors	AOR of postpartum depression: women who had a pre-pregnancy check-up or treatment for depression vs. women who did not have a check-up or treatment: 2.09, 95% CI 1.24–3.55.
Rockhill et al. (2015) [85]	AI/AN, Urban and rural	Retrospective cohort study of AI/AN women to understand relationship between pre-pregnancy BMI and gestational weight gain on birthweight	Included 8663 AI/AN women who participated in the Pregnancy Risk Assessment Monitoring System 2004–2011	AI/AN race, maternal age, parity, any diabetes during pregnancy, smoking during pregnancy, gestational weight gain, and state of residence	AOR of high-birthweight baby (4000 g+): overweight vs. normal weight women: 1.27, 95% CI 1.01–1.59; obese vs. normal weight women: 1.57, 95% CI 1.25–1.96.
Seal and Broome (2013) [86]	American Indian, Not stated/unclear	Cross-sectional survey of American Indian mothers of full-term infants to understand relationships between pre-pregnancy BMI, feeding practices, and infant growth	Included 98 American Indian mothers of infants of 14–20 weeks who visited an outpatient clinic	Crude	No significant associations between pre-pregnancy BMI and birthweight.
Tabet et al. (2017) [91]	AI/AN, Urban and rural	Retrospective cohort study of women, including AI/AN women, to understand risk factors for late preterm birth (34–36 weeks’ gestation)	Included 171,573 women <20 years, including 2459 AI/AN women, with singleton first births at 34–44 weeks’ gestation in 2012	Age, marital status, health insurance, prenatal care, infant sex, gestational weight gain, and infection	AOR of spontaneous late preterm birth: underweight vs. normal weight women: 1.42, 95% CI 0.68–3.01 (not significant); overweight vs. normal weight women: 0.69, 95% CI 0.38–1.25 (not significant); obese vs. normal weight women: 0.81, 95% CI 0.45–1.47 (not significant). AOR of medically indicated late preterm birth: underweight vs. normal weight women: 0.80. 95% CI 0.10–6.25 (not significant); overweight vs. normal weight women: 2.08, 95% CI 0.92–4.69 (not significant); obese vs. normal weight women: 1.18, 95% CI 0.41–3.39 (not significant).
Tiwari et al. (2021) [93]	AI/AN, Not stated/unclear	Retrospective cohort study of women, including AI/AN women, using data from the Obstetrical Care Outcomes Assessment Program database to understand the relationship between pre-pregnancy overweight/obesity and pregnancy complications	Included 72,697 women, including 978 AI/AN women, with singleton live births from 2014 to 2018 in Washington State	Maternal age, parity, delivery hospital, health insurance, substance abuse, nicotine use, and alcohol use	ARR of pre-eclampsia: overweight/obese vs. normal weight women: 5.24, 95% CI 1.92–14.30. ARR of caesarean section: overweight/obese vs. normal weight women: ARR 1.57, 95% CI 1.07–2.31. There was no association between overweight/obese status and gestational diabetes.
Wojcicki et al. (2015) [94]	Alaska Native, Urban and rural	Retrospective cohort study of Alaskan women, including Alaska Native women, to understand maternal and child factors associated with obesity at age 3	Included 833 women, including 277 Alaska Native women, who completed the Risk Assessment Monitoring System 2005–2006 and the Childhood Understanding Behaviors Survey (CUBS) 2008–2009	Maternal age, child witnessing abuse, geographical location, participating in WIC nutrition program antenatally, breastfeeding, child fried potato consumption, child fruit juice consumption, maternal chew/spit tobacco use, and income	AOR obese child at age 3 years: maternal pre-pregnancy obesity vs. no obesity: 1.87, 95% CI 0.94–3.72 (not significant).
Zamora-Kapoor et al. (2016) [95]	AI/AN, Urban and rural	Retrospective cohort study of AI/AN women matched with non-Indigenous women to understand risk factors for pre-eclampsia	Included 7194 AI/AN women with singleton live first births in Washington State 2003–2013, matched with a sample of 63,886 white women	N/A	Risk of pre-eclampsia by BMI category: underweight: 4.6%; normal weight: 5.6%; overweight: 9.2%; obese: 13.7%. No statistical analysis was performed on these variables.

Abbreviations: CI = confidence interval, SD = standard deviation, OR = odds ratio, AOR = adjusted odds ratio, APR = adjusted prevalence ratio, ARR = adjusted relative risk, HR = hazard ratio, GDM = gestational diabetes mellitus, GWG = gestational weight gain. BMI categories are defined as follows: underweight = <18.5, normal weight = 18.5–24.9, overweight = 25–29.9, class I obese = 30–34.9, class II obese = 35–39.9, class III obese = 40+.

**Table 3:** Studies describing development, implementation, or evaluation of preconception health interventions.

Author and Date	Setting (Indigenous Pop, Geographical Remoteness)	Intervention	Study Description	Participants/Sample	Key Results
**Australia**					
Bower et al. (2016) [40]	Aboriginal, Urban and rural	Mandatory folic acid bread fortification	Repeated cross-sectional study of Aboriginal men and non-pregnant women and analysis of WA Register of Developmental Anomalies to compare folate status and Neural Tube Defects in the Aboriginal population before and after bread fortification	Included 95 Aboriginal men and non-pregnant women age 16–44 years (subsequent measurement)	Mean red cell folate concentrations increased by 41% in males and 49% in females after mandatory fortification (mean difference before and after fortification: males 129 ng/mL, 95% CI 81–177; females 186 ng/mL, 95% CI 139–233). The prevalence ratio for Neural Tube Defects in the Aboriginal population for the post-fortification period (2010–2014) was 0.32 (95% CI 0.15–0.69) compared with 1980–2009.
Kennedy et al. (2021) [45]	Aboriginal and Torres Strait Islander, Urban and rural	Preconception and pregnancy multibehavioural change app for Aboriginal women (MAMA-EMPOWER)	Mixed methods study including 1. interviews with Aboriginal women, 2. workshop with Aboriginal women, 3. app trial with Aboriginal women, and 4. user Mobile Application Rating Scale survey of Aboriginal women to develop and test MAMA-EMPOWER	Phase 1 interviews: 8 urban Aboriginal women, Phase 2 workshop: 6 Aboriginal women, Phase 3 app trial: 16 urban and regional Aboriginal women, and Phase 3 survey: 5 Aboriginal women	Participants informed the content, functionality, and user experience of the app. User MARS survey ratings were highest for information (mean score 3.80 out of 5, SD 0.77) and aesthetics (mean score 3.87, SD 0.74), while functionality (mean score 3.0, SD 0.73), engagement (mean score 3.2, SD 1.08), and subjective quality (mean score 3.2, SD 0.95) had lower scores. Qualitative feedback indicated that the developed app was acceptable; however, functionality was problematic.
Munns et al. (2016) [48]	Aboriginal, Rural	Preconception health activities as part of a community antenatal program	Mixed methods review, including interviews and focus groups with staff and partner agencies and quantitative reporting data, to understand acceptability and satisfaction with an Aboriginal Community Antenatal Program which included preconception services	Included 22 staff and representatives from partner agencies	Participants raised a range of organisational factors, staff factors, cultural issues, and interagency issues which affected implementation.
**Canada**					
Bains et al. (2014) [52]	Inuit and Inuvialuit, Rural	Twelve-month community-based diet and lifestyle intervention aimed at Inuit and Inuvialuit women of childbearing age (Healthy Foods North)	Controlled pre–post evaluation	Included 136 women of 19–44 years from 6 Inuit and Inuvialuit communities in Arctic Canada (79 exposed and 57 in control group)	ANCOVA analysis demonstrated a positive impact of the intervention on vitamin A (pre–post change for intervention group vs. control= 558.23 mcg, 95% CI 179.86–936.59) and vitamin D (pre–post change for intervention group vs. control = 89.23 IU, 95% CI 3.86–174.60). There was no significant change in energy, sugar, or fat consumption or other nutrients (folate, calcium, or iron).
**New Zealand**					
Mallard and Houghton (2014) [58]	Māori, Not stated/unclear	Mandatory iodine bread fortification	Cross-sectional survey of postpartum women to understand iodine intake	Included 723 women who had given birth in birthing centres across New Zealand in March–April 2011, including 93 Māori women	Following fortification, an estimated 5% of Māori women were below the Estimated Average Requirement for iodine preconception (95% CI 0–11%), compared with 15% prior to fortification (95% CI 3–27%).
Mallard et al. (2012) [57]	Māori, Not stated/unclear	Mandatory folic acid bread fortification (modelled)	Cross-sectional survey of postpartum women to understand bread intake prior to and during pregnancy and model effects of proposed mandatory folic acid fortification	723 women who had given birth in birthing centres across New Zealand in March-April 2011, including 93 Māori women	Assuming mandatory bread fortification, mean preconception folic acid intake from bread for Māori women would be 173.1 mcg/day (95% CI 144.8–201.5). However, 47.1% of Māori women would not be consuming the target of an additional 140 mcg of folic acid from bread (95% CI 35.6–58.7%).
**United States**					
Charron-Prochownik et al. (2023) [61]	AI/AN, Urban and rural	‘Stopping GDM’—gestational diabetes preconception risk reduction and counselling intervention for AI/AN teenagers at high risk of GDM	Analysis of baseline data from a GDM risk reduction intervention for at-risk AI/AN teens, ‘Stopping GDM’, to explore associations between GDM risk reduction awareness, knowledge, health beliefs, and behaviours	Included 149 female AI/AN young women, 12–24 years old, and their mothers (or adult female caregivers) who participated in Stopping GDM	At baseline, mothers had greater knowledge (measured as a percentage of questioned answered correctly) of GDM prevention than their daughters (mean score on a 0 to 100 scale: mothers 49.2, SD 20.9, daughters: 20.9, SD 20.4, *p* < 0.001) and perceived greater susceptibility than their daughters (mean score on a 0 to 20 scale: mothers 8.9, SD 4.0, daughters 7.0, SD 3.8, *p* < 0.001). Daughters reported moderate levels of self-confidence (self-efficacy) in their ability to engage in healthy living (mean score on an 8 to 80 scale: 48.2, SD 13.9) but low mean scores on healthy eating (mean score, 9.0, SD 5.6) and physical activity (mean score: 3.4, SD 2.2). Mothers were more likely to initiate GDM communication with daughters than vice versa (mean score: mothers 2.9, SD 0.9, daughters 2.3, SD 1.3, *p* < 0.001)
Deutsch et al. (2021) [63]	AI/AN, Not stated/unclear	Brief alcohol-exposed pregnancy reduction program (OST CHOICES)	Qualitative analysis of alcohol exposure prevention strategies by AI/AN women	Included 160 AI/AN women of 18–42 years who participated in OST CHOICES	Common prevention strategies included positive social supports and avoiding negative or alcohol-involved social environments. Other strategies used circular logic (i.e., drinking less to reduce drinking). Traditional and Western cultural strengths were identified as important resources, although many participants did not have a cultural resource strategy.
Gorman et al. (2013) [65]	AI/AN, Not stated/unclear	Brief online program for screening and prevention of prenatal alcohol use	Qualitative focus groups and interviews with AI/AN women and key informants to modify a brief online program for screening and prevention of prenatal alcohol use	Included 15 AI/AN women of childbearing age and 6 key informants	Participants reported that the program and format were acceptable and raised issues of confidentiality, the importance of family and culture, and tailored content and recommended including information on the negative impacts of alcohol on child health.
Hanson et al. (2011) [68]	American Indian, Not stated/unclear	Alcohol-exposed pregnancy prevention and awareness media campaign	Mixed methods study including focus groups with American Indian women, elders, and community members and a survey of American Indian women to develop and evaluate an alcohol-exposed pregnancy prevention and awareness media campaign in the Northern Plains	Included 5 American Indian women, 18–44, 10 elder women, and 25 community members, who participated in focus groups and 119 American Indian women of 18–44 years who completed a survey	Focus group participants identified the importance of including traditional language and themes as well as positive messaging in the campaign. Overall, 91.6% of survey participants agreed that the campaign increased their knowledge of Foetal Alcohol Syndrome, and 71.8% agreed that the campaign decreased their drinking (no further statistical analysis conducted).
Hanson et al. (2013) [69]	American Indian, Rural	Telephone-based alcohol-exposed pregnancy reduction program	Longitudinal survey (baseline, 3, 6, 9, and 12 months)	Included 231 non-pregnant American Indian women of 18–44 years who participated in a phone-based intervention modified from Project CHOICES program	The proportion of participants at risk of alcohol-exposed pregnancy reduced from 54% at baseline to 20% at 12 months (difference between baseline and all other visits *p* < 0.001). Alcohol consumption decreased across all behavioural measures over the intervention duration (average change −26% to −17%, 99% CIs −41% to −7%), and the proportion of participants reporting no birth control decreased from baseline to 3 months (29–10%, *p* < 0.001).
Hanson et al. (2015) [70]	American Indian, Rural	Group-based brief alcohol-exposed pregnancy reduction program (CHOICES Group)	Mixed-methods study, including survey of non-pregnant American Indian women of 18–44 years and observations to evaluate group mode of delivery for CHOICES Group	Included 33 non-pregnant American Indian women of 18–44 years who participated in the pilot of CHOICES Group	Participants reported that sessions positively engaged members (median engagement score session 1: 4.8/6, session 2: 4.4/6), had low levels of conflict (median conflict score session 1: 0.6/6, session 2: 0.4/6), and had average levels of avoidance of personal responsibility (median avoidance score session 1: 3.7/6, session 2: 3.3/6). Observations found that group leaders possessed some motivational interview skills, with improvement needed in other motivational interviewing and leadership skills.
Hanson et al. (2017) [71]	American Indian, Urban and rural	Brief alcohol-exposed pregnancy reduction program (OST CHOICES)	Longitudinal survey (baseline and 3 and 6 months post-intervention) of non-pregnant American Indian women to evaluate OST CHOICES	Included 99 non-pregnant American Indian women of 18–44 years who participated in OST CHOICES	All women were at risk at baseline. This dropped to 25.4–47.2% at 3 months and 18.1–66.3% at 6 months depending on modelling assumptions. For those who reduced AEP risk, this was most commonly due to using effective birth control (3 months: 67.7, 6 months: 61.5%) rather than lowering binge drinking (3 months: 9.8%, 6 months: 20.0%) or both behaviours (3 months: 22.6%, 6 months: 18.5%).
Jensen et al. (2016) [74]	American Indian, Urban and rural	Brief alcohol-exposed pregnancy reduction program (OST CHOICES)	Qualitative interviews with health and social service professionals and focus groups with American Indian men and women to inform the expansion of OST CHOICES	Included 25 health and social service professionals and 58 American Indian men and women	Participants suggested a focus on younger people through schooling and other channels and raised the role of family and culture in preventing alcohol-exposed pregnancies.
Lowrey et al. (2019) [77]	American Indian, Urban and rural	Brief alcohol-exposed pregnancy reduction program (OST CHOICES)	Qualitative written responses of American Indian women to understand participant experiences using a transtheoretical model	Included 203 American Indian women who participated in OST CHOICES	Participants’ responses indicated progression through stages of change from participating in the program. Participants reported that their children, education, and work were motivators to decrease unhealthy behaviours. Common barriers to behaviour change were reported and included stress and temptation to drink socially.
Montag et al. (2015) [78]	AI/AN, Not stated/unclear	Online Screening, brief intervention, and referral to treatment through alcohol-exposed pregnancy prevention intervention	Randomised controlled trial with survey data at baseline and 1, 3, and 6 months post-intervention) of AI/AN women	Included 247 AI/AN women of 18–45 years who received an online alcohol-exposed pregnancy prevention intervention or usual treatment	Both groups decreased self-reported risky drinking behaviour (risk of alcohol-exposed pregnancy *p* < 0.001 at 6 months post-intervention), with no significant difference between groups.
Montag et al. (2017) [79]	AI/AN, Not stated/unclear	Peer-to-peer screening, brief intervention and referral to alcohol-exposed pregnancy prevention intervention	Qualitative focus groups and interviews with American Indian women, community leaders, Tribal Elders, and health staff to inform adaption of online screening, brief intervention, and referral to treatment alcohol-exposed pregnancy prevention intervention to a peer-to-peer, motivational interviewing format	Included 10 focus groups (n = 54) and 3 interviews with American Indian women of 18–45 years, community leaders, Tribal Elders, and health staff	Participants identified adaptions to make the intervention appropriate for the peer-to-peer format, culturally appropriate, and relevant for the target audience. Participants suggested making changes to content about assessing alcohol consumption, assessing why women drink, and assessing why women do not drink.
Moore et al. (2019) [80]	AI/AN, Urban	‘Stopping GDM’—gestational diabetes preconception risk reduction and counselling intervention for AI/AN teenagers at high risk of GDM	Qualitative focus groups with AI/AN adolescent females at risk of gestational diabetes (and their mothers) about their awareness and understanding of GDM and reproductive health to inform the adaption of a preconception counselling and diabetes education intervention	Included 13 AI/AN adolescent females at risk of gestational diabetes and their mothers	Participants identified a lack of awareness and knowledge of GDM, a need for pregnancy planning and culturally responsive GDM and reproductive health resources, and importance of empowerment to promote positive reproductive health behaviours.
Moore et al. (2021) [81]	AI/AN, Not stated/unclear	‘Stopping GDM’—gestational diabetes preconception risk reduction and counselling intervention for AI/AN teenagers at high risk of GDM	Qualitative focus groups with AI/AN tribal leaders and health care administrators to inform the development of Stopping GDM	Included 12 AI/AN tribal leaders and health care administrators who attended the 2015 National Indian Health Board Conference in Washington, DC	Participants made recommendations regarding effective communication with AI/AN youth (avoiding directive language, including health information, using relevant imagery and language), the importance of family and community engagement, and incorporating diverse traditional AI/AN cultural values and practices.
Nadeau et al. (2020) [83]	AI/AN, Urban	‘Stopping GDM’—gestational diabetes preconception risk reduction and counselling intervention for AI/AN teenagers at high risk of GDM	Pre–post study of AI/AN daughters and their mothers to evaluate Stopping GDM prior to a randomised controlled trial	Included 11 AI/AN daughters at risk of GDM and their mothers recruited through an urban Indian health program	Mean knowledge relating to diabetes prevention increased pre-intervention to post-intervention (mothers: 70% to 80%, daughters: 45% to 58%), as did reproductive health and GDM knowledge (mothers: 65% to 92%, daughters: 34% to 71%). Daughters’ self-efficacy increased for healthy living (47.2 ± 11.1 to 56.6 ± 14.3, possible score 8–80) and pregnancy planning (57.0 ± 9.2 to 66.4 ± 17.7, possible score 12–120). Satisfaction for the materials was moderately high to very high.
Richards and Mousseau (2012) [84]	American Indian, Not stated/unclear	Preconception health educational intervention aimed at American Indian high school students	Randomised controlled evaluation	Included 58 American Indian high school students who attended a summer high school residential academic program (28 exposed and 30 in control group)	The intervention group scored higher than the non-intervention group in overall preconception health knowledge (96% vs. 90%, *p* = 0.03) and obesity knowledge post-intervention (44% vs. 33%, *p* = 0.01). Other health areas (smoking, alcohol, diabetes, contraception) were not significant.There were significant changes in obesity and diabetes knowledge for the intervention group from pre- to post-intervention (obesity: 28% vs. 48%, *p* = 0.01, diabetes: 36% vs. 72%, *p* = 0.02). Other health areas (general preconception health, smoking, alcohol, contraception) were not significant.
Shreshtha et al. (2018) [87]	American Indian, Urban and rural	Brief alcohol-exposed pregnancy reduction program (OST CHOICES)	Mixed-methods study, including behavioural survey and qualitative analysis of drinking behaviours and attitudes of non-pregnant American Indian women participating in OST CHOICES	Included 264 non-pregnant American Indian women of 18–44 years who participated in OST CHOICES	Mean alcohol self-efficacy was −0.04 (range: −23 to 24). Participants reported positive aspects of drinking including escaping from problems, socializing, and relaxation. Negative aspects included impact on families and domestic violence.
Stotz et al. (2019) [88]	AI/AN, Urban	‘Stopping GDM’—gestational diabetes preconception risk reduction and counselling intervention for AI/AN teenagers at high risk of GDM	Qualitative interviews and focus groups with AI/AN women with type 2 diabetes or a history of GDM to inform the development of Stopping GDM	Included 5 AI/AN women with type 2 diabetes or a history of GDM	Participants raised a lack of knowledge on GDM and risk factors, importance of culture and family, suggestions for communication with AI/AN girls to reduce risk of GDM (open non-judgemental conversation, using stories, and connecting to culture), and the emotional impact of GDM diagnosis.
Stotz et al. (2021) [89]	AI/AN, Not stated/unclear	‘Stopping GDM’—gestational diabetes preconception risk reduction and counselling intervention for AI/AN teenagers at high risk of GDM	Qualitative interviews and focus groups with AI/AN women, AI/AN girls and their mothers, health care providers and administrators, AI/AN tribal leaders, and health system administrators to understand how stakeholders understand perceptions on food and nutrition in terms of gestational diabetes risk reduction for AI/AN adolescent girls	Included 5 AI/AN women with a history of GDM, 14 AI/AN girls at risk of GDM and their mothers (n = 11), 16 health care providers and administrators, 12 AI/AN tribal leaders, and health system administrators	Participants raised that AI/AN women were not aware of how healthy nutrition and healthy weight gain during pregnancy were linked to reducing risk of GDM, the need for education on the role of nutrition and weight management in GDM risk reduction, and challenges of healthful eating before and during pregnancy for AI/AN women.
Stotz et al. (2023) [90]	AI/AN, Not stated/unclear	‘Stopping GDM’—gestational diabetes preconception risk reduction and counselling intervention for AI/AN teenagers at high risk of GDM	Secondary analysis of randomised controlled trial of a GDM risk reduction intervention for at-risk AI/AN teens, ‘Stopping GDM’, to explore food insecurity as a potential moderator of the effect of Stopping GDM on healthy eating behaviours and self-efficacy	Included 149 female AI/AN young women, 12–24 years old, and their mothers (or adult female caregivers) who participated in Stopping GDM	At 3 months post-intervention, the intervention group had increases in times eating vegetables per week (mean change on a scale of 0 to 24: intervention = 1.0, 95% CI −0.2 to 2.2, control = −0.9 95% CI −2.0 to 0.2, *p* = 0.022) and fruit (mean change on a scale of 0 to 6: intervention 0.5, 95% CI 0.6 to 0.9, control −0.3, 95% CI −0.8 to 0.2, *p* = 0.015), whereas the control group had declines. Food insecurity did not moderate the group by time interaction for healthy eating self-efficacy (*p* ≥ 0.05) but did moderate the group by time interaction for times drinking soda (*p* = 0.004) and days eating breakfast (*p* = 0.013).
Terry et al. (2020) [92]	AI/AN, Urban and rural	‘Stopping GDM’—gestational diabetes preconception risk reduction and counselling intervention for AI/AN teenagers at high risk of GDM	Qualitative interviews and focus groups with health professionals to inform the adaption of a preconception counselling and diabetes education intervention for AI/AN teens	Included 16 health professionals with expertise in AI/AN youth, adolescent health, and gestational diabetes	Participants felt that the content would be appropriate for AI/AN teens and their mothers or another adult. Participants made recommendations regarding effective communication with AI/AN teens, including culturally appropriate images, using non-directive language, and education with a community focus. Concerns included: socioeconomic issues that affect AI/AN people such as food and housing insecurity, abuse, and historical trauma.

**Table 4:** Studies of uptake of preconception care and preconception health knowledge and attitudes.

Author and Date	Setting (Indigenous Population, Geographical Remoteness)	Study Description	Participants/Sample	Key Results
**Australia**				
Gilbert et al. (2021) [43]	Aboriginal and Torres Strait Islander, Rural	Mixed methods study including Aboriginal and Torres Strait Islander and non-Indigenous youth to understand Aboriginal and Torres Strait Islander youth preconception awareness and knowledge and to co-design a preconception health resource	Included 4 Aboriginal and Torres Strait Islander and 3 non-Indigenous youth of 18–25 years living in Darwin and employed as trainees or undergraduate research assistant cadets, who participated in a youth working group and 24 residents of the NT of 16–22 years who completed a survey	Participants of consultation meetings identified an interest in fertility and a need for more information on lifestyle factors associated with infertility. Participants were interested in learning more about preconception health, including regarding the opposite sex, and using information that incorporates current local knowledge and world views. Of survey respondents, 45% had a pre-existing understanding of preconception health. Optimising lifestyle behaviours prior to pregnancy was perceived as important for women (88%), but less so for men (67%).
Griffiths et al. (2020) [44]	Aboriginal, Rural	Retrospective audit of medical records of Aboriginal women to understand preconception health care delivery	Included 127 Aboriginal women who attended a remote Australian Aboriginal Community Controlled Health Service and who had at least one pregnancy from 2011 to 2018.	In total, 121/177 (68%) of confirmed pregnancies had received preconception care prior to the pregnancy. Sexually transmissible infection screening (71%) was the most common care delivered, followed by folic acid prescription (57%) and smoking cessation support (43%). Nutrition and weight (36%), alcohol and illicit substances (26%), chronic disease management (17%), and vaccinations (12%) were less common preconception care interventions. Preconception care was usually patient-initiated (63%), conducted by a nurse or Aboriginal Health Worker (59%) and increased significantly over the audit period (number of consultations *p* = 0.003).There were no differences in the likelihood of receiving preconception care by age group, pregnancy outcome, gestation at first antenatal visit, parity, diabetes or albuminuria status, BMI, or smoking behaviour. Younger women were less likely to be screened for chronic diseases (linear trend across age groups *p* < 0.01).
**New Zealand**				
Tromop-van Dalen et al. (2021) [59]	Māori, Not stated/unclear	Mixed methods study including 1. review of clinical letters for women with heart disease and 2. survey of Wellington Hospital cardiologists to understand frequency of preconception counselling and barriers	Included 199 women of 15–52 years with heart disease who attended Wellington Hospital cardiology clinics in 2015–2020 (clinical letter review) and 16 Wellington Hospital cardiologists, who completed a survey	In total, 32% of Māori women had documented contraception and preconception counselling discussions.Barriers to discussing contraception were time limitations, cultural barriers, presence of family members, not considered, considered outside of expertise.
**United States**				
Lammers et al. (2017) [76]	Native American, Rural	Cross-sectional survey of women, including Native American women, to understand awareness and access to preconception health care	Included 868 women of 18–45 years, including 258 Native American women, who were receiving community health services from the South Dakota Department of Health	In total, 79.1% of Native American women reported receiving 1+ preconception health care interventions.

## Data Availability

All data generated or analysed during this study are included in this published article and its supplementary information files.

## Supplementary Materials

The following supporting information can be downloaded at: https://www.mdpi.com/article/10.3390/ijerph21030345/s1, File S1. Search strategies in CINAHL and PubMed.
