# Supplementary material for: Preconception Health of Indigenous Peoples in Australia, Canada, New Zealand, and the United States: A Scoping Review
Source: Int J Environ Res Public Health. 2024 Mar 14;21(3):345. doi: 10.3390/ijerph21030345 (PMC10969840; doi:10.3390/ijerph21030345)
Supplement: Supplementary file 1 [file ijerph-21-00345-s001.zip › ijerph-2776983-supplementary.docx]

**File S1. Search strategies in CINAHL and PubMed**

## Search strategy in CINAHL

*Conducted 31 August 2022, updated 25 June 2023*

| **#** | **Query** | **Limiters/Expanders** | **Last Run Via** |
| --- | --- | --- | --- |
| S7 | S5 AND S6 | Limiters - Published Date: 20100101-20231231; Language: English | Interface - EBSCOhost Research Databases |
|  |  | Expanders - Apply equivalent subjects | Search Screen - Advanced Search |
|  |  | Search modes - Boolean/Phrase | Database - CINAHL Complete |
| S6 | S3 OR S4 | Expanders - Apply equivalent subjects | Interface - EBSCOhost Research Databases |
|  |  | Search modes - Boolean/Phrase | Search Screen - Advanced Search |
|  |  |  | Database - CINAHL Complete |
| S5 | S1 OR S2 | Expanders - Apply equivalent subjects | Interface - EBSCOhost Research Databases |
|  |  | Search modes - Boolean/Phrase | Search Screen - Advanced Search |
|  |  |  | Database - CINAHL Complete |
| S4 | AB "Aboriginal Australia*" OR "Indigenous Australia*" OR "Torres Strait Island*" or "First Nation*" or “First people*” or “First Australian*” or “First Canad*” or “First America*” or Maori* or “Pacific Island*” or “Pacific People*” Inuit* or Eskimo* or Metis* or Amerindian* or "Native Americ*" or "Alaska Native*" or “Native Alaska*” or “Native Canad*” | Expanders - Apply equivalent subjects | Interface - EBSCOhost Research Databases |
|  |  | Search modes - Boolean/Phrase | Search Screen - Advanced Search |
|  |  |  | Database - CINAHL Complete |
| S3 | (MH "Maori") OR (MH "First Nations of Australia") OR (MH "Native Americans") OR (MH "Aboriginal Canadians") OR (MH "Alaska Natives") OR (MH "First Nations of Canada") OR (MH "Aboriginal Australians") | Expanders - Apply equivalent subjects | Interface - EBSCOhost Research Databases |
|  |  | Search modes - Boolean/Phrase | Search Screen - Advanced Search |
|  |  |  | Database - CINAHL Complete |
| S2 | AB pre-pregnan* or prepregnan* or "pre pregnan*" or pre-concept* or preconcept* or "pre concept*" or pre-gravid or pregravid or “consider* pregnan*” or “contemplat* pregnan*” or "before pregnan*" or "prior to pregnan*" or “before concept*” or “prior to concept*” or interconcept* or inter-concept* or interpregnan* or inter-pregnan* or inter-natal* or internatal or peri-concept* or periconcept* or “reproductive age” or “reproductive years” or “childbearing age” or “childbearing years” | Expanders - Apply equivalent subjects | Interface - EBSCOhost Research Databases |
|  |  | Search modes - Boolean/Phrase | Search Screen - Advanced Search |
|  |  |  | Database - CINAHL Complete |
| S1 | (MH "Prepregnancy Care") | Expanders - Apply equivalent subjects | Interface - EBSCOhost Research Databases |
|  |  | Search modes - Boolean/Phrase | Search Screen - Advanced Search |
|  |  |  | Database - CINAHL Complete |

## Search strategy in PubMed

*Conducted 31 August 2022, updated 25 June 2023*

((("Preconception Care"[MeSH Terms] AND "english"[Language]) OR (("pre pregnan*"[Title/Abstract] OR "prepregnan*"[Title/Abstract] OR "pre pregnan*"[Title/Abstract] OR "pre concept*"[Title/Abstract] OR "preconcept*"[Title/Abstract] OR "pre concept*"[Title/Abstract] OR "pre-gravid"[Title/Abstract] OR "pregravid"[Title/Abstract] OR "before pregnan*"[Title/Abstract] OR "prior to pregnan*"[Title/Abstract] OR "before concept*"[Title/Abstract] OR "prior to concept*"[Title/Abstract] OR "interconcept*"[Title/Abstract] OR "inter concept*"[Title/Abstract] OR "interpregnan*"[Title/Abstract] OR "inter pregnan*"[Title/Abstract] OR "internatal"[Title/Abstract] OR "peri concept*"[Title/Abstract] OR "periconcept*"[Title/Abstract] OR "reproductive age"[Title/Abstract] OR "reproductive years"[Title/Abstract] OR "childbearing age"[Title/Abstract] OR "childbearing years"[Title/Abstract]) AND "english"[Language] AND "english"[Language])) AND "english"[Language] AND (((("indians, north american"[MeSH Terms] OR "Native Hawaiian or Other Pacific Islander"[MeSH Terms]) AND "english"[Language]) OR (((("aboriginal australia*"[Title/Abstract] OR "indigenous australia*"[Title/Abstract] OR "torres strait island*"[Title/Abstract] OR "first nation*"[Title/Abstract] OR "first people*"[Title/Abstract] OR "first australian*"[Title/Abstract] OR "first canad*"[Title/Abstract] OR "first america*"[Title/Abstract] OR "maori*"[Title/Abstract] OR "pacific island*"[Title/Abstract] OR "pacific people*"[All Fields]) AND "inuit*"[Title/Abstract]) OR "eskimo*"[Title/Abstract] OR "metis*"[Title/Abstract] OR "amerindian*"[Title/Abstract] OR "native americ*"[Title/Abstract] OR "alaska native*"[Title/Abstract] OR "native alaska*"[Title/Abstract] OR "native canad*"[Title/Abstract]) AND "english"[Language])) AND "english"[Language])) AND ((english[Filter]) AND (2010:2023[pdat]))
